# Validity and Reliability of the New Portable Metabolic Analyzer PNOE

**DOI:** 10.3389/fspor.2019.00024

**Published:** 2019-09-10

**Authors:** Yiannis E. Tsekouras, Konstantinos D. Tambalis, Stavros E. Sarras, Athanasios K. Antoniou, Peter Kokkinos, Labros S. Sidossis

**Affiliations:** ^1^Department of Nutrition and Dietetics, School of Health Science & Education, Harokopio University, Athens, Greece; ^2^Department of Kinesiology and Health, Rutgers University, New Brunswick, NJ, United States; ^3^Department of Cardiology, Veterans Affairs Medical Center, Washington, DC, United States; ^4^Georgetown University School of Medicine, Washington, DC, United States; ^5^Department of Medicine, Robert Wood Johnson Medical School, Rutgers University, New Brunswick, NJ, United States

**Keywords:** indirect calorimetry, breath-by-breath analysis, validity, reliability assessment, portable metabolic measuring system

## Abstract

Assessment of the oxygen and carbon dioxide content of expired air during exercise is critical for determining cardiorespiratory status. The purpose of this study was to compare the new portable metabolic analyzer PNOE with COSMED – Quark CPET, a previously validated stationary metabolic cart.

**Methods:** A total of 22 subjects (17 male and 5 female) aged 32.3 ± 11.1 years took part in the study. Breath by breath gas exchange was measured by both devices during a four-stage incremental protocol on a cycle ergometer. On a separate day, 10 participants repeated the trial to assess the reliability of the PNOE metabolic cart.

**Results:** Strong correlations were obtained in VO_2_ (*r* = 0.98, *p* < 0.001), VCO_2_ (*r* = 0.98, *p* < 0.001), VE (*r* = 0.98, *p* < 0.001), and RQ (*r* = 0.91, *p* < 0.001), between the two devices. Bland-Altman plots revealed a mean difference of 34.0 ± 118 ml/min and 36.4 ± 110 ml/min in VO_2_ and VCO_2_ analysis, respectively. There were no significant differences in VO_2_, VCO_2_, VE, or RQ between the two devices. Intraclass correlation coefficient was high between the two trials for VO_2_ (*r* = 0.98, *p* < 0.001), VCO_2_ (*r* = 0.98, *p* < 0.001), VE (*r* = 0.99, *p* < 0.001), and RQ (*r* = 0.93, *p* < 0.001).

**Conclusions:** Our data indicate that the portable metabolic cart PNOE can accurately determine respiratory gases over a wide range of exercise intensities, in healthy individuals, in a controlled laboratory setting.

## Introduction

Cardiorespiratory function has been extensively studied over the years by measuring oxygen uptake (VO_2_) and carbon dioxide production (VCO_2_) in exercising individuals (Kaminsky et al., 2017). Assessment of VO_2_ in elite athletes and recreationally active participants has provided individualized information concerning training adaptations (Capostagno et al., 2019). Moreover, in clinical conditions, determination of VO_2_ and respiratory quotient (RQ) have provided information on the energy cost and substrate utilization while running, walking or cycling, thus optimizing the dose of exercise to improve general health indices (Swainson et al., 2019). Most of these assessments have been performed in laboratory conditions and have incorporated stationary metabolic carts, under the supervision of specialized and trained personnel. This is essential to obtain reliable information, however the process may become labor-intensive and sometimes cost-prohibitive (Parvataneni et al., 2009). Such limitations prevent the widespread application of the assessment and highlight the necessity to design valid and reliable metabolic units that will be portable and practical to use outside the laboratory settings. In this way, respiratory variables may become readily available to the sports scientist to monitor cardiorespiratory fitness, in the long-term and prescribe exercise plans for health and performance (da Cunha et al., 2011).

Until recently, several portable metabolic units have been developed and studied to accurately determine energy metabolism in outdoor and indoor activities. Amongst these units, the Oxylog (Ballal and Macdonald, 1982), the Cosmed K2 to K5 versions (Littlewood et al., 2002; Schrack et al., 2010; Perez-Suarez et al., 2018), AeroSport TEEM 100 (Novitsky et al., 1995), the CORTEX X1 and Meta Max (Macfarlane and Wong, 2012) and the Oxycon Mobile metabolic system (Rosdahl et al., 2010) have been assessed for their validity and reliability, providing evidence for their strengths and weaknesses. From these validation studies it becomes evident that portable devices tend to be less accurate compared to the stationary metabolic carts (Brehm et al., 2004) and there is still need for developing small, portable, albeit valid and reliable metabolic analyzers, to quantify respiratory variables under real-life conditions.

Therefore, the aim of this study was to address the accuracy and reliability of the new metabolic cart PNOE (ENDO Medical, Palo Alto, CA), in measuring VO_2_, VCO_2_, RQ, and VE in the expired air of exercising individuals, during a stationary cycling protocol. It is hypothesized that the utility of the PNOE portable metabolic analyzer is comparable to that of a valid and reliable stationary metabolic unit (COSMED - Quark CPET) (Nieman et al., 2013).

## Methodology

### Subjects

Twenty-two subjects (17 men, 5 women), from a cycling club, volunteered to participate in the study. Physical characteristics are summarized in Table 1. All subjects were recreationally active and participated in organized cycling sessions at an average frequency of 3 to 4 times per week. Subjects were asked to maintain their normal diet and refrain from strenuous or unaccustomed exercise for the 24 h preceding the tests. They were also instructed to fast for at least 4 h and to abstain from ingestion of alcohol for 48 h before the tests. All subjects signed a written informed consent, prior to their participation in the study. The study was approved by the Harokopio University ethical committee for research involving human participants. All procedures were in accordance with the Helsinki Declaration of 1975 as revised in 1996.

**Table 1:** Subject characteristics in the validity and the reliability study.

	**Validity study**		**Reliability study**	
	***Males* (*n* = 17)**	***Females* (*n* = 5)**	***Males* (*n* = 6)**	***Females* (*n* = 4)**
Age (years)	31.9 ± 10.5	32.8 ± 13.6	32.3 ± 11.0	35.2 ± 14.4
Height (cm)	177.1 ± 6.0	168.4 ± 9.2	179.2 ± 6.6	171.8 ± 6.2
Weight (kg)	80.1 ± 8.2	64.0 ± 13.1	77.0 ± 4.9	66.9 ± 10.3
Body Mass Index (kg/m^2^)	25.1 ± 2.3	22.5 ± 3.6	23.9 ± 2.6	22.7 ± 4.2

*Values are expressed as mean ± SD*.

### Study Design and Experimental Procedure

Body stature (cm) and body mass (kg) were measured, without shoes. Subjects performed the validation exercise protocol after performing a 5 min warmup (mild pedaling at 50 W) on a stationary cycling ergometer (Ergoline 800 s, Bitz, Germany) and a five min stretching. The Ergoline ergometer was selected, as it maintains steady power production in case that changes in revolutions per min (rpm) are detected. Thus, it was ensured that a constant load was applied at all times. The validation protocol was performed at a constant rate of 60–65 rpm during four exercise intensities (four stages). Each stage lasted for five min, starting from 75W and increasing by 25 W or 50 W according to the subjects perceived fitness, as estimated by the visual inspection of the heart rate response and the Borg Scale score. The intensity levels were classified to reflect relatively light, moderate and vigorous cardiorespiratory demands, according to the American College of Sports Medicine (ACSM) (Garber et al., 2011). Similar exercise intensity range has been previously selected by others (Rosdahl et al., 2010; Perez-Suarez et al., 2018). Respiratory variables of VO_2_, VCO_2_, RQ and VE were calculated using values from the final minute of each five-min stage. During this timeframe, heart rate coefficient of variation (CV) was measured to be < 5%. Additionally, five 10-s averages were taken for these respiratory values and a CV below 10% was confirmed.

**Figure 1:**
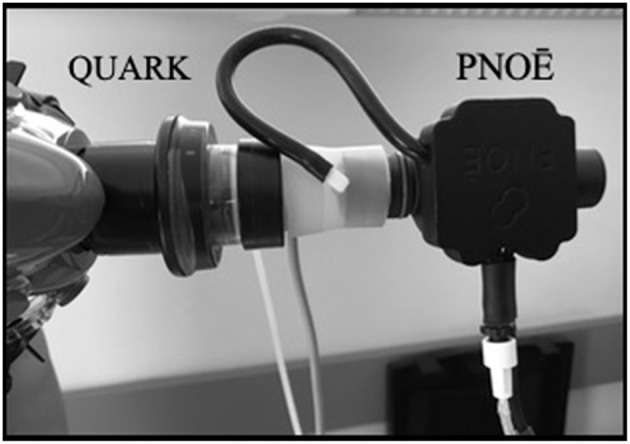
The In-line set up between Quark CPET and PNOE flow sensors.

Measurements were made in-line (sequential gas sampling) with the COSMED - Quark CPET (Quark CPET) and PNOE (ENDO Medical, Palo Alto, CA) (Figure 1). The Quark CPET system acted as a reference standard, as it has been shown to be a valid metabolic system in its breath by breath mode (Nieman et al., 2013). The dead space between the two flow sensors has been quantified and accounted for in the calculations. The sequential in-line set up may provide advantages over repetitive assessment trials on different days since it eliminates the subject's biological day to day variation. The in-line connection of the two systems (Quark CPET and PNOE) was tested at a metabolic simulator (Relitech, Netherlands) prior to this validation study. A metabolic simulator was chosen for this test as it can generate fully reproducible conditions in terms of VE (Rosdahl et al., 2010). These tests were conducted to make sure that the in-line connection of the flow sensors (from the Quark CPET system and the PNOE system) does not affect ventilation (VE). In particular, the turbine of the Quark CPET system was connected with an adaptor to the MEMS (micro electro mechanical systems) based hot-film anemometer flow sensor of the PNOE system and the setup was tested at the metabolic simulator, in three different settings of simulated breathing frequency (BF = 20, 40, and 60 strokes per minute). VE in the metabolic simulator was set at 1 L throughout the experiments.

Once the in-line connection was tested, a second experiment was conducted with exactly the same procedure (BF = 20, 40, 60 strokes per minute), by mounting the turbine flow sensor of Quark CPET system to the metabolic simulator. The relative error between the in-line connection of the two flow sensors and the turbine of the Quark CPET system was 1.18%. The error of 1.18% lies within the technical specifications of the turbine flow sensor, as reported by the manufacturer (Cosmed, Rome), which is 1.5% when measuring VE.

The same procedure was followed with the PNOE system. VE measured by its standalone flow sensor was compared to the VE obtained from the in-line system. The relative error between the two measurements was 1.15%. Again, this error lies within the technical specification reported by the manufacturer of the PNOE system (ENDO Medical, Palo Alto, CA). These results demonstrate that the in-line connection of the two systems does not affect their operation and it can be used in this validation study. Such protocols have been successfully tested in the past (Prieur et al., 2003). Calibration of flow was performed with the two systems in line, using multiple pumps of a 3 L calibration syringe. Both devices were calibrated under specific gas concentrations after each measurement.

The reliability of the PNOE device was assessed by measuring gas exchange variables on ten randomly selected participants, on a separate day, at least 2 days after the first measurement. During this second visit, subjects were requested to adhere to similar nutritional habits before the repetition exercise protocol. The same experimental setup was followed in both visits.

### Description of the Portable Metabolic Cart PNOE

PNOE is a newly developed portable metabolic cart that was designed to measure gas exchange under laboratory and field conditions. The unit operates on lithium batteries and weighs approximately 800 g. The device is composed of a single housing (120 × 110 × 45 mm, height, width, length, respectively), fastened to a shoulder harness and carried by the subject throughout exercise. The subject wears a suitable facemask and breathes through the flow sensor that is a MEMS-based hot film anemometer flow sensor. The advantage of this technology lies in the fact that it does not contain moving parts and is considered to be a stable construction to use in a portable device. Heart rate and respiratory gases are transmitted via telemetry. PNOE operates on a breath-by-breath mode that continuously measures volume and determines expired gas concentrations simultaneously. It measures VO_2_ via an open-circuit indirect calorimetry technique by assessing pulmonary gas exchange at the mouth and the nose. The components of the unit include an electrochemical O_2_ analyzer and an infrared CO_2_ analyzer. The standard Hans Rudolph mask was used with the mouthpiece and the head support (Hans Rudolph Inc., Kansas City, MO, USA).

## Statistical Analysis

Results were normally distributed and are presented as mean values and standard deviation (means ± sd). Statistical significance was accepted at the 5% level (*P* ≤ 0.05). Power analysis was performed from a pilot sample (*N* = 10 subjects) to determine sample size. The effect size (ES) in this study was 0.39 and is considered to be medium according to Cohen's criteria. With alpha = 0.05 and power = 0.80, the projected sample size needed (estimated by GPower3.1 software), would be *N* = 23, for between group comparisons (Faul et al., 2007). Our sample size of *N* = 22 (alpha = 0.05) provides a power of 0.78. Student's paired samples *t* tests were used to compare differences between the variables measured by Quark CPET and PNOE. Pearson correlation coefficients were used to define agreement between the two metabolic carts. Intraclass correlation coefficient (ICC) was used to assess test retest reliability of the PNOE. A 95% confidence interval (CI) was used to describe the variety in the ICC. Bland Altman plots of the difference between Quark CPET and PNOE were plotted against the average of the two measures (Bland and Altman, 1986). All analyses were performed using the Statistical Package for the Social Sciences (SPSS), Version 23.0, for Windows.

## Results

**Figure 2:**
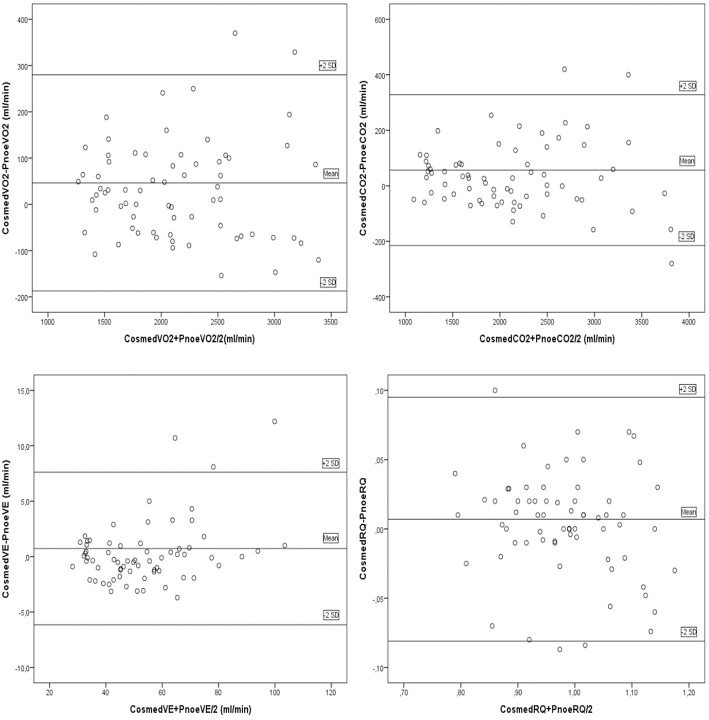
Bland Altman plots for the comparison between the Quark CPET and PNOE devices for the following variables: **(A)** VO_2_, **(B)** VCO_2_, **(C)** VE, and **(D)** RQ.

Fifteen males and seven females successfully completed the validity test. On a separate day, a sub-group of them consisting of six males and four females, conducted the repeatability test. Data on VO_2_, VCO_2_, VE, and RQ, for each intensity stage and comparisons between the two devices, are presented in Table 2. No significant differences were reported between the two metabolic carts, regarding respiratory variables, at all intensity stages. Mean percentage differences between the two devices in VO_2_, VCO_2_, VE and RQ were 2.2, 2.3, 1.4, and 0.6%, respectively. Pearson correlation demonstrates a high degree of agreement, for all the respiratory variables, between the two devices (0.93 < Pearson < 0.99, *P* < 0.001) (Table 3). Test-retest measures show strong reliability for the PNOE device (ICC's > 0.90) (Table 3). Bland – Altman plots for the relationships between the two metabolic carts are shown in Figure 2. A high degree of agreement is observed for VO_2_, VCO_2_, VE, and RQ between Quark CPET and PNOE measures across all intensity stages, as revealed by a mean difference of 34.0 ± 118 ml/min and 36.4 ± 110 ml/min in VO_2_ and VCO_2_ analysis, respectively. All subjects, except from two cases in VO_2_ and three cases in VCO_2_, VE and RQ, lie within the 2 standard deviation acceptance range of the Bland Altman plots.

**Table 2:** Variables measured by the two metabolic carts for each stage.

**Variables**	**Quark *(Mean ± SD)***	**PNOE*(Mean ± SD)***	**Mean difference *(Mean ± SD)***	**% Diff**	***P*-value**
**VO**_**2**_ (ml*min^**−1**^**)**					
Stage 1	1,522 ± 173	1,484 ± 122	36.2 ± 108	4.4	0.091
Stage 2	1,973 ± 262	1,947 ± 229	26.2 ± 114	1.3	0.291
Stage 3	2,450 ± 368	2,412 ± 350	39.2 ± 124	2	0.192
Stage 4	2,924 ± 447	2,889 ± 456	34.9 ± 121	1.5	0.173
Mean	2,195 ± 606	2,161 ± 605	34.0 ± 118	2.3	0.107
**VCO**_**2**_ (ml*min^**−1**^**)**					
Stage 1	1,358 ± 150	1,282 ± 153	76.5 ± 117	5.6	0.137
Stage 2	1,914 ± 256	1,878 ± 233	36.9 ± 118	1.9	0.230
Stage 3	2,491 ± 423	2,443 ± 415	48.7 ± 141	2	0.291
Stage 4	3,072 ± 525	3,006 ± 561	66.2 ± 175	2.2	0.642
Mean	2,209 ± 338	2,152 ± 341	57.1 ± 138	2.6	0.207
VE (ml*min^**−1**^**)**					
Stage 1	36.0 ± 6.0	35.2 ± 4.8	0.8 ± 3.4	2.2	0.105
Stage 2	47.7 ± 6.7	47.8 ± 5.4	−0.1 ± 2.9	−0.2	0.221
Stage 3	62.4 ± 11.4	61.6 ± 10.2	0.7 ± 3.5	1.1	0.501
Stage 4	76.3 ± 16.6	74.6 ± 14.4	1.8 ± 4.0	2.3	0.411
Mean	55.6 ± 10.2	54.8 ± 8.7	0.8 ± 3.5	1.4	0.309
**RQ**					
Stage 1	0.90 ± 0.08	0.88 ± 0.09	0.017 ± 0.05	1.94	0.172
Stage 2	0.97 ± 0.07	0.96 ± 0.07	0.007 ± 0.04	0.76	0.278
Stage 3	1.02 ± 0.07	1.01 ± 0.09	0.002 ± 0.04	0.21	0.376
Stage 4	1.04 ± 0.07	1.04 ± 0.09	−0.002 ± 0.05	−0.18	0.903
Mean	0.98 ± 0.07	0.98 ± 0.08	0.006 ± 0.04	0.64	0.201

*The level of significance is based on the Student's paired-sample t tests, for the mean difference of the variables measured by Quark CPET and PNOE*.

**Table 3:** Correlation coefficient data for the validity and the reliability test between Quark CPET and PNOE.

	**Validity test**	**Test-retest reliability**
	***Pearson***	***ICC (95% CI)***
VO2 (ml/min)	0.98^**^	0.98 (0.96–0.99)^**^
VCO2 (ml/min)	0.98^**^	0.98 (0.96–0.99)^**^
VE (ml/min)	0.98^**^	0.99 (0.97–0.99)^**^
RQ	0.93^**^	0.91 (0.83–0.99)^**^

ICC, Intraclass Correlation Coefficient; CI, Confidence Interval.

** *P < 0.001*.

## Discussion

This is the first study to examine the validity and reliability of the PNOE metabolic cart, during a four-stage incremental cycling protocol. Comparison was performed against a stationary device, Quark CPET, which has been previously assessed for its validity and reliability, in performing respiratory gas analysis in healthy adults (Nieman et al., 2013). Data analysis suggests that PNOE is capable to accurately and reliably determine VO_2_, VCO_2_, VE and RQ, under laboratory conditions.

The importance of undertaking validation and repeatability studies in gas analysis devices is important to obtain representative cardiorespiratory evaluations, not only in athletic but also in clinical populations (Meyer et al., 2005). The practicality of the process will facilitate sports scientists in providing individualized exercise prescription (Sartor et al., 2013). The protocol of the present study involved cycling over a wide range of exercise intensities ranging from easy to vigorous (approximately 50–98% of participants peak heart rate), according to the ACSM Position Statement (Garber et al., 2011). At this range of exercise intensities, the PNOE device provided a valid assessment of respiratory gasses for all subjects. This is highlighted by the visual inspection of the Bland Altman plots showing significant agreement between the two metabolic carts (Quark CPET and PNOE), in the measurements of VO_2_, VCO_2_, VE, and RQ. A serial method (in-line setup) of assessment has been selected, where all expired gasses sequentially pass through both measuring devices to reduce variance and avoid the additive effect of day to day error from the participants (biological error) and the metabolic cart (technical error). This setup was assessed for its interference with the measuring process, on a gas simulator and its effect was found to be negligible (CV of < 1.15%, which lies below the instruments technical specifications).

Previous validation studies, including portable metabolic carts, often demonstrate deviations in VO_2_ that tend to become greater as exercise intensity increases. Minor overestimation by approximately 3–7% has been reported in the range of low to moderate intensity exercise (McLaughlin et al., 2001; Brehm et al., 2004; Crouter et al., 2006; Ross et al., 2019) for indoor and outdoor exercise, respectively. A slightly higher (5–9%), between device difference in VO_2_ has been reported during high intensity or near maximum intensity exercise (Rosdahl et al., 2010; Perez-Suarez et al., 2018).

In the present study, the fourth stage of exercise involved intense cycling, at an intensity over 85% of subjects' estimated VO_2_peak. Such exercise lies beyond the anaerobic threshold, as becomes evident from the high RQ values (RQ>1) (Elmer and Toney, 2018). In this last stage, mean difference in VO_2_ values between the two devices was not significantly different. CV for submaximal to near maximal cycling VO_2_ measurement has been estimated to be close to 5% (Crouter et al., 2006) and within device difference in the present study lies within this limit (2.3%). Furthermore, regarding the VCO_2_ validity, the level of similarity lies within the range of acceptance as observed in Bland Altman plots. Thus, for this range of exercise intensity, the RQ, representing the ratio of VCO_2_ to VO_2_ may accurately serve as an indicator of energy substrate utilization and could effectively be used to address energy metabolism during exercise (McClave et al., 2003). This is supported by the highly similar RQ values between the two metabolic carts.

It has been suggested that the use of turbine flow sensors [technology that until recently, most portable metabolic devices effectively incorporate in their systems (Guidetti et al., 2018)], may underestimate respiratory data, especially at very high flow rates (Rosdahl et al., 2010). High intensity exercise involves significant hyperventilation, and this may affect flow rate recording. Furthermore, most portable metabolic devices use a fixed value of 31° C in the algorithm to calculate VE (Rosdahl et al., 2010) and this may not always be the case in high breathing frequencies, where air flow around the sensor is cooler. The PNOE device uses a MEMs based hot-film anemometer sensor to detect flow on a subject's breath (Cruz et al., 2007). Hot-film anemometer consists of a wire that is constantly electrically heated, by the system battery, to maintain a specific temperature. Temperature decrements, due to the flow of the respiratory gasses, are interpreted as increments in resistance and in this way, it is possible for the system to detect minor changes on the flow rate and provide accuracy in VE and hence VO_2_, VCO_2_ and RQ measures.

In the present study, the reliability trial involved the repetition of the exercise protocol on a different day under the same laboratory conditions. These results support satisfactory repeatability of the PNOE device, comparable to the stationary Quark CPET device, as suggested by the relatively high ICC values. Similar data have been reported by others (Rosdahl et al., 2010).

## Limitations

All measurements were conducted under controlled laboratory conditions with stable temperature and humidity, on a stationary cycle ergometer. This was imperative to minimize the external environmental effects for the repeatability trials. Our findings may not be applicable in other settings, such as performing activities that require significant bouncing and in outdoor environmental conditions involving temperature/humidity deviations from the standard laboratory settings. Future studies may provide evidence for the efficacy of the device during outdoor activities.

In summary, our data indicate that the PNOE portable metabolic cart is as accurate as a state-of-art stationary metabolic cart, capable of measuring respiratory variables with precision, during a wide range of exercise intensities, under laboratory conditions. Furthermore, the use of the MEMs based hot film anemometer sensor may become a promising alternative to the widely used turbine flow sensor, in the breath by breath systems and provide direct measurements of inspired and expired air volume.

## Data Availability

The datasets generated for this study are available on request to the corresponding author.

## Ethics Statement

The studies involving human participants were reviewed and approved by the Harokopio University ethical committee for research involving human participants. The patients/participants provided their written informed consent to participate in this study.

## Author Contributions

LS and PK conceived and designed the experiments. SS, AA, and LS performed the experiments and data collection. LS, PK, and KT analyzed the data. YT and KT wrote the first draft of the manuscript. All co-authors edited and proofread the manuscript and approved the final version.

### Conflict of Interest Statement

LS served as scientific advisor to ENDO Medical, Palo Alto, CA, the manufacturer of the PNOE system, from November 2016 to March 2018. The remaining authors declare that the research was conducted in the absence of any current commercial or financial relationships that could be construed as a potential conflict of interest.
